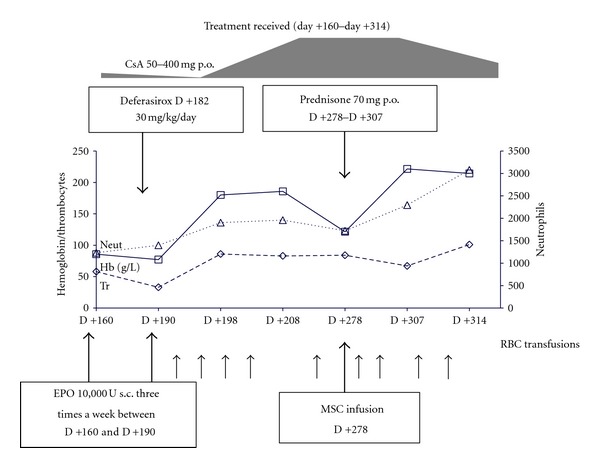# Rapid Recovery from Chronic PRCA by MSC Infusion in Patient after Major ABO-Mismatched alloSCT

**DOI:** 10.1155/2012/862721

**Published:** 2012-06-17

**Authors:** Vera Sergeevicheva, Irina Kruchkova, Elena Chernykh, Ekaterina Shevela, Alexander Kulagin, Andrey Gilevich, Igor Lisukov, David Sergeevichev, Vladimir Kozlov

**Affiliations:** ^1^Federal State Budgetary Institution “Research Institute of Clinical Immunology”, Russian Academy of Medical Sciences Siberian Branch, Novosibirsk 630099, Russia; ^2^St. Petersburg Pavlov State Medical University, Saint Petersburg 197022, Russia; ^3^Federal State Institution “Academician EN Meshalkin Novosibirsk Research Institute of Circulation Pathology” of the Ministry of Public Health and Social Development, Novosibirsk 630055, Russia

## Abstract

Pure red cell aplasia (PRCA) is a rare complication in recipients of allogenic stem cell from ABO incompatible donors. It is characterized by reticulocytopenia and by an absence of red cell cell precursors in the bone marrow. Despite close isohemagglutinins monitoring and standard immunosupressive treatment in these patients prolong PRCA are still associated with severe transfusion dependence. We report the case of a 31 yr old male patient who underwent HLA-matched ABO-mismatched allo-SCT and developed resistance PRCA despite conventional immunosupressive therapy and prophylaxis cotrasplantation of bone marrow derived MSC at day 0. He responded dramatically to therapy with adipose tissue derived mesenchymal stem cells from HSC donors and continued to be transfusion-independent and AML-disease free. This method of the PRCA therapy of deserves further investigation.

Allogeneic stem cell transplantation (allo-SCT) is one of the curative treatment options for patients with acute myeloid leukemia (AML). However, immunological mismatch can increase complications such as GVHD and PRCA. Delayed donor red cell engraftment and partial red cell aplasia (PRCA) occur in cases of major ABO mismatch between donor and recipient due to inhibition of the donor's erythroid progenitors by isohemagglutinins produced by residual plasma cells. After discovery of the high immunomodulatory effect of mesenchymal stem cells (MSCs), it was demonstrated that MSC infusion is a promising method of prophylaxis and treatment for post-BMT complications, such as severe acute GVHD and PRCA in clinical practice [1–3].

This paper presents the rapid erythroid recovery following MSC infusion in a patient with resistant PRCA due to ABO-incompatible alloSCT. This study was in accordance with the Helsinki Declaration, and it was approved by the Local Ethic Committee of the Institute of Clinical Immunology SB RAMS. The informed written consent was signed by the patient and donor before the treatment.

The patient was a 31-year-old man with acute myeloid leukemia, M5 variant in the first remission who underwent haematopoietic stem cell transplantation from his HLA-matched sister on February 14, 2008. Conditioning therapy consisted of busulfan 16 mg/kg p.o. and cyclophosphamide 120 mg/kg. The transplanted PBSC allograft contained 4.4 × 10*6/kg CD34+ cells with less than 15 mL RBC in this stem cell product.

There was a major ABO mismatched between the donor (A+) and recipient (0+). Graft versus host disease prophylaxis included cyclosporine A (CsA) started from day −1 and short courses of methotrexate on days +1, +3, +6, and +11. Also to prevent GVHD, we used a culture-expanded population of the donor's MSC at a dose of 1.2 × 10*6/kg, derived from a small marrow aspirate sample which was cotransplanted on day 0. Neutrophil engraftment was seen on day +16. However, mixed chimerism was confirmed by VNTR-PCR-based method, and complete donor chimerism was observed only on day +190.

On day +62, the patient was still dependent on RBC transfusions with no donor red cell chimerism, and anti-A isohemagglutinin titer was 1:8 (this was the highest detected level). Due to profound reticulocytopenia, lasting more than 60 days after allo-SCT (max reticulocyte count was 5 × 10^9^/L), and the lack of erythroid precursors on bone marrow examination, pure red cell aplasia was diagnosed. The patient showed no evidence of GVHD, viral infection, hemolysis, onset of alloantibodies, or relapse.

The treatment of PRCA employed several therapeutic approaches (Figures 1, and 2). On day +28, the patient was given erythropoietin (EPO) at a dose of 10.000U three times a week for a month with rapid CsA tapering. On days +65 and +72, we initiated the treatment with anti-CD20 antibody (rituximab) at a dose of 500 mg once a week, and two doses were given without effect. Oral prednisolone therapy on day +96 was also ineffective. The patient also failed to respond to the second 4-week course of EPO therapy. Elevated ferritin level (2560 ng/mL) was detected, and chelation therapy by Deferasirox at 30 mg/kg/day was started on day +182.

On day +237 the patient was still dependent on blood transfusions (4–6 RBC units per month) resulting in iron overload, and there was a high risk of infection transmission. It was decided to recruit his sister again as MSC donor. MSC was isolated from a 50 mL bone marrow and a 40 mL subcutaneous adipose tissue as previously described [4, 5]. Culture media did not contain any xenobiomaterial: fetal calf serum was exchanged with 5% human allogenic platelet lysate [6]. High surface expression of CD73, CD90, and CD105 was determined on the culture-expanded MSCs by flow cytometry. MSCs were confirmed to be negative for markers CD3, CD34, CD16, CD20, CD14, HLA-DR. The cells were cultured negative for bacteria, mycoplasma, and fungi before infusion.

On day +278, the patient received MSC at the dose 0.9 x 10*6/kg. At the same time, we diagnosed the first symptoms of chronic GVHD in the patient: weight loss above 5 kg during the last month, lichen planus in the mouth, and xerostomia. Simultaneously with MSC infusion, the patient started therapy with CsA (6 mg/kg/day) and prednisolone (1 mg/kg/day). At 4 weeks after MSC infusion (day +307), the patient showed the appearance of erythroid precursor (12%) on bone marrow examination and improvement of cGVHD symptoms. By day +314, the patient had a dramatic increase of hemoglobin from 6.7 g/dl on day +307 to 10.1 g/dl with reticulocytes at 95 × 10*9/L. Conversion from O- to A-blood group was observed on day +327.

Therefore, in this case, we used MSC transplantation twice. The first infusion as aGVHD prophylaxis on day 0 was associated with absence of aGVHD, but we watched prolonged mixed chimerism and development of PRCA after myeloablative conditioning regimen. We hypothesized that a second infusion of donor's MSC may aid the complete recovery of erythropoiesis. This effect can be explained by the suppressive influence of MSC on donors T–cell and/or on recipient's residual B cells [7–9]. The MSC-mediated immunosuppression mainly acts through the secretion of soluble molecules that are induced or upregulated following crosstalk with target cells. Among these factors, indoleamine 2,3-dioxygenase (IDO) has consistently been reported [10]. We have evaluated 2.3 IDO expression by the donor's MSCs to explain its potential mechanism of immune tolerance induction. BM-derived donors MNCs were negative for IDO expression, whereas IDO was detected in adipose-derived MSC constitutively. But the suggestion that the effect of immune tolerance upregulation is due to only IDO expression requires further evaluation as the effect could also be due to other compounds expressed by the MSCs (PGE-2, TGFb, NO, and HLA-G5) and others [9]. 

To prevent GVHD in this present case, we used BM-derived donor MSC as a single infusion at day 0, simultaneously with donor hematopoietic stem cells (according to our local clinical trial). This patient had no GVHD but developed PRCA. The reason of PRCA development is persistence of recipient antibody-producing plasma cells which survived after myeloablative conditioning and were able to produce isoagglutinins. Theoretically, B–cell proliferation must have been restrained under MCSs as well. But the immunosuppressive effect of MSC is transient, and PRCA symptoms appeared at a later time. It is well known that T–cell activation is strongly inhibited even at low concentration of MCS, but the effect of MSC on B cells appeared to be influenced by the relative concentrations *in vitro*, though their effect on B cells may require T cells. We used MCS again at 9 months after transplantation. This interaction of MSC with other immune cells resulted in resolution of PRCA. For treatment of PRCA, we used adipose tissue-derived MSC which can have distinct properties of different kind. Moreover, MCS may intensify the immunosuppressive effect of standard therapy with prednisone and CsA [11]. On conclusion, we propose that the source of MSC, timing of infusion, and interactions of MSCs with various immune cells in the microenvironment can influence differences in outcome of MSCs infusion and require further researches. Future studies would be required in order to clarify the potential role of MSC in the treatment of immune-mediated complication after allogenic SCT. The patient is warrantably alive and disease-free at January of 2012.

## Figures and Tables

**Figure 1:**
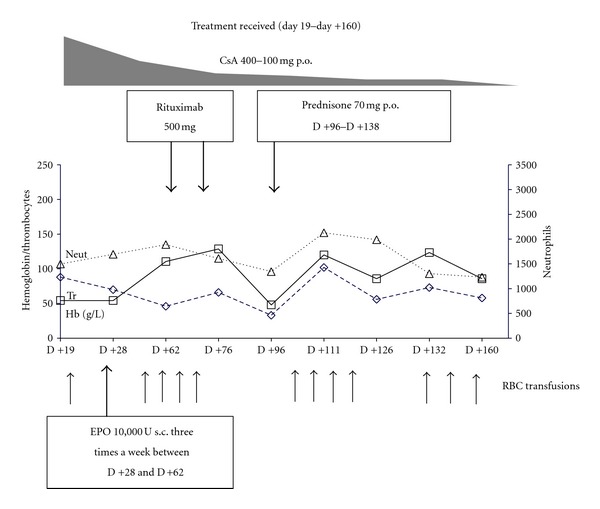


**Figure 2:**